# A novel mutation in the tyrosine kinase domain of *ERBB2 *in hepatocellular carcinoma

**DOI:** 10.1186/1471-2407-6-278

**Published:** 2006-12-06

**Authors:** Tanios Bekaii-Saab, Nita Williams, Christoph Plass, Miguel Villalona Calero, Charis Eng

**Affiliations:** 1 Division of Hematology and Oncology, Department of Internal Medicine, Comprehensive Cancer Center, The Ohio State University, Columbus, Ohio, USA; 2 Department of Pharmacology, The Ohio State University, Columbus, Ohio, USA; 3 Division of Human Genetics, Department of Internal Medicine, Comprehensive Cancer Center, The Ohio State University, Columbus, Ohio, USA; 4 Department of Molecular Virology, Immunology and Medical Genetics; Human Cancer Genetics Program, Comprehensive Cancer Center, The Ohio State University, Columbus, Ohio, USA; 5 Genomic Medicine Institute and Taussig Cancer Center, Cleveland Clinic Foundation, Cleveland, Ohio, USA

## Abstract

**Background:**

Several studies showed that gain-of-function somatic mutations affecting the catalytic domain of *EGFR *in non-small cell lung carcinomas were associated with response to gefitinib and erlotinib, both *EGFR*-tyrosine kinase inhibitors. In addition, 4% of non-small cell lung carcinomas were shown to have *ERBB2 *mutations in the kinase domain. In our study, we sought to determine if similar respective gain-of-function *EGFR *and *ERBB2 *mutations were present in hepatoma and/or biliary cancers.

**Methods:**

We extracted genomic DNA from 40 hepatoma (18) and biliary cancers (22) samples, and 44 adenocarcinomas of the lung, this latter as a positive control for mutation detection. We subjected those samples to PCR-based semi-automated double stranded nucleotide sequencing targeting exons 18–21 of *EGFR *and *ERBB2*. All samples were tested against matched normal DNA.

**Results:**

We found 11% of hepatoma, but no biliary cancers, harbored a novel *ERBB2 *H878Y mutation in the activating domain.

**Conclusion:**

These newly described mutations may play a role in predicting response to EGFR-targeted therapy in hepatoma and their role should be explored in prospective studies.

## Background

Mutations in the protein-kinase enzyme family, such as the epidermal growth factor receptor (*EGFR*, *ERBB2*), found in human cancers are being investigated as promising targets for the development of novel antitumor therapies.*EGFR *is the first described member of a family of related transmembrane receptor tyrosine kinases. It is comprised of the following four related receptors: *EGFR *itself (*ERBB1 *or HER1), *ERBB2 *(HER2/*neu*), *ERBB3 *(HER3) and *ERBB4 *(HER4). *ERBB *receptors are composed of an extracellular ligand-binding domain, a transmembrane segment, and an intracellular protein tyrosine kinase domain. These receptors trigger downstream signaling pathways that are complex and multilayered. Deregulation of those *ERBB *receptors can lead to malignant transformation. These receptors form either homo- or heterodimeric complexes which provides amplification and diversification [1]. Heterodimerization of the *ERBB2 *and *EGFR *is associated with a more robust signaling than homodimerization [2]. Several studies showed that gain-of-function somatic mutations affecting the catalytic domain (specifically the ATP binding site, exons 18–21) of *EGFR *in non-small cell lung carcinomas were strongly associated with response to gefitinib and erlotinib, both related *EGFR*-tyrosine kinase inhibitors (TKI) [3-5]. More recently, a number of studies reported the presence of *ERBB2 *mutations located in the kinase domain (exons 19 and 20) in non-small cell lung carcinomas (NSCLC) that could potentially result in the activation of the tyrosine kinase activity of the receptor protein [6-9]. In addition to NSCLC, mutations in the *ERBB2 *kinase domain were described in gastric, colorectal, and breast cancers [10,11]. In our study, we sought to determine if similar respective gain-of-function *EGFR *and *ERBB2 *mutations were present in hepatoma and biliary cancers to determine the potential for *ERBB *-targeted therapy.

Hepatoma is the most common malignant tumor of the liver with a clear rising incidence in the number of cases in the United States, and is largely attributed to the increase in hepatitis C related liver disease [12]. Cancers of the biliary tract are the second most common primary hepatobiliary cancer [13]. There is no satisfactory treatment available for patients with hepatobiliary cancers and chemotherapy has been extremely disappointing.

The poor prognosis of patients with hepatoma and biliary cancers in addition to the lack of satisfactory therapy for advanced cases indicates a need for more effective therapeutic options. *EGFR *signaling is implicated in both hepatic and biliary carcinogenesis. Studies indicate that *EGFR *is expressed in up to 85% and 80% of hepatoma and biliary cancers, respectively [14], and EGF might be required for the growth of those cells [15,16]. *ERBB2 *is also expressed in a significant number of hepatoma and biliary cancers acting as an independent prognostic factor and a major contributor to carcinogenesis [17-21]. Recently, a multi-center phase 2 study looked at the efficacy and tolerability of erlotinib in advanced hepatoma and biliary cancers with encouraging results [22,23]. In hepatoma, the reported PFS at 6 months of 32% with disease control (PR + SD) of 59% for a median duration of 4 months. The median overall survival (OS) was 13 months. In biliary cancers, the reported PFS at 6 months was 25% with disease control (PR + SD) of 55% for a median duration of 5.4 months. The median OS was 9 months.

## Methods

In our study, we extracted genomic DNA from 40 hepatoma (18) and biliary cancers (22) samples, and 44 adenocarcinoma of the lung, the latter as a positive control for mutation detection. All hepatobiliary samples were paraffin-embedded while all lung samples were frozen. Hepatoma and biliary cancers were subjected to laser capture microdissection as previously described (Kurose K et al. Hum Mol Genet 2001) to enrich the neoplastic component. We subjected those samples to PCR-based semi-automated double stranded nucleotide sequencing, per routine of the Eng lab [24,25]. Exons 18–21 (exon-intron junctions and flanking intronic regions) of *EGFR *were amplified using the primers and conditions described by Lynch et al [3] and those of *ERBB2 *were amplified using the primers and conditions described by Stephens et al [6]. Double stranded sequencing was performed with the ABI-3700. Mutations or variants were confirmed in both directions as well as confirmed by a second independent PCR reaction. All samples were tested against matched germline DNA derived from the related normal organ. Our samples were acquired on anonymised archived materials without personal identifiers, on an exempt protocol approved by the Ohio State University Institutional Review Board.

## Results

**Figure 1:**
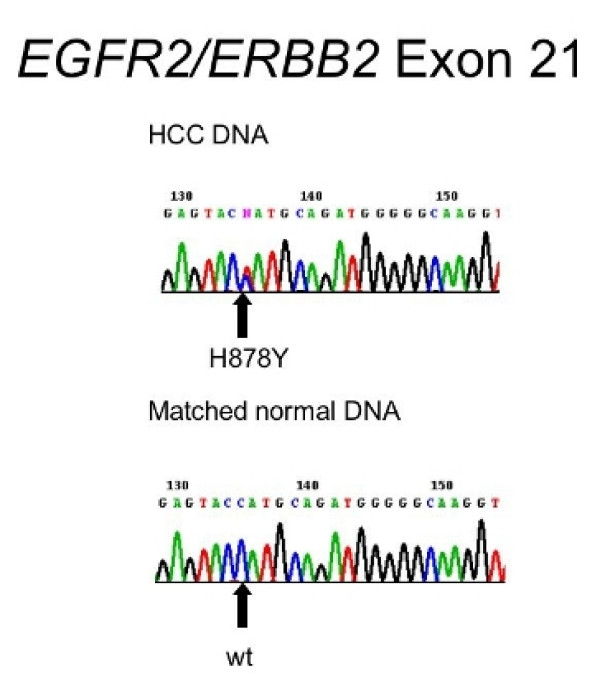
Somatic missense mutation, H878Y, found in genomic DNA from hepatoma (top chromatogram) but not in corresponding normal tissue (bottom panel).

All hepatoma and biliary cancers samples were negative for gain-of-function somatic mutations affecting the catalytic domain of the *EGFR *gene. Power calculations suggest that our negative results predict that no more than 3% of hepatoma or biliary cancers would eventually be found to carry such mutations. As a positive control for mutation detection, we sequenced *EGFR *exons 18–21 in 44 adenocarcinoma of the lung and found 2 (5%) with somatic delE746-A750 mutations, previously reported to be associated with gefitinib responsiveness. In contrast, 2 (11%) of the 18 hepatoma had a single novel somatic H878Y (CAT to TAT; c.2632C > T) mutation in exon 21 of *ERBB2 *(figure 1), while none of the biliary cancers were found to have somatic mutations. None of the matched normal tissue was found to have somatic mutations for *EGFR *or *ERBB2 *found in the tumors. These are different from the reported mutations in non-small cell lung carcinomas. The H878Y missense mutation occurring in the activation domain of *ERBB2 *alters a basic hydrophilic residue to an acidic hydrophilic residue, and so is predicted to affect function.

## Discussions and Conclusion

The lack of effective treatment for hepatobiliary tumors indicates a need for more effective therapeutic options. Emerging targeted therapies including EGFR inhibitors are offering promise in a variety of malignancies including hepatobiliary tumors. From this study, we conclude that gain-of-function somatic mutations affecting the catalytic domain of *EGFR *as described in non-small cell lung carcinomas have a predicted low yield in hepatoma and biliary cancers. A recently published study showed the lack of somatic mutations in EGFR tyrosine kinase domain in 100 hepatocellular carcinomas confirming our findings [26]. In contrast, we showed that 11% of hepatocellular carcinomas tested have an *ERBB2 *mutation occurring in the activation domain. None were found in biliary cancer specimens. Given the availability of agents interfering with *ERBB2 *signaling and the established role of *ERBB2 *in the *EGFR *signaling pathway, we speculate that these newly described mutations may play a role in predicting response to EGFR-targeted therapy in hepatoma in predicting response to agents that target *ERBB2 *and/or *EGFR *in hepatoma. Interestingly, one such agent, lapatinib (a dual *EGFR*/*ERBB2 *TKI), was recently reported to have activity in hepatomas (TGCR = 40%) but not in biliary cancers (TGCR = 26%) [27]. Cohen et al showed that in head and neck cancers although responses to gefitinib or erlotinib were not linked to kinase mutations of *EGFR*, one single response case was actually linked to a mutation in exon 20 of *ERBB2 *establishing the potential predictive value of such mutations on responses to agents that target *ERBB2 *and/or *EGFR *[28]. In conclusion, our findings suggest that mutations in the tyrosine kinase domain of *ERBB2 *in hepatoma may underlie responsiveness to agents that target *ERBB2 *and/or *EGFR*. This should trigger similar analysis as part of correlative studies in prospective studies.

## Competing interests

The author(s) declare that they have no competing interests.

## Authors' contributions

TBS conceived the study in collaboration with CE, was involved in the coordination of the study and drafted the manuscript. NW carried out the technical aspects of the mutation analysis of EGFR/ERBB2. CP made substantial contributions to conception and design and interpretation of data. MVC participated in the design of the study and was involved in revising the manuscript for intellectual content. CE conceived the study in collaboration with TBS, supervised the conduct of the study and helped draft the manuscript. All authors read and approved the final manuscript.

## Pre-publication history

The pre-publication history for this paper can be accessed here:
